# Understanding the relationship between hypothermia and surgical site infections (SSI’S) in order to improve recording and management of hypothermia pre, intra and post operatively

**DOI:** 10.1186/1753-6561-5-S6-O56

**Published:** 2011-06-29

**Authors:** SE Smith, J Wayland

**Affiliations:** 1 Clinical Safety and Quality, Melbourne, Australia; 2 Infection Control, St. Vincent's & Mercy Private Hospital Melbourne, Melbourne, Australia

## Introduction / objectives

As part of the of the hospital “No Harm” strategy, hypothermia knowledge, assessment and treatment was identified as being a gap in the prevention of surgical site infections. The aim of this project was to create an awareness of hypothermia management, understand the importance of preventing hypothermia and measure the overall impact of hypothermia on SSI rate.

## Methods

Baseline awareness surveys and hypothermia management audits were completed. A program was designed that was inclusive of patients, family and staff and was aimed at creating awareness and knowledge surrounding the importance of hypothermia prevention in the surgical patient. Patients were provided with preadmission information about keeping warm and staff educated regarding the human impact of the adverse effects and consequences of hypothermia. Hypothermia management at one of the hospital sites’ theatres had already been instigated. Findings and project outcomes were gathered with the view of implementing at the other sites.

## Results

Results demonstrate an increased awareness of hypothermia treatment and management in the surgical patient. There was a reduction in time spent in recovery as patients’ temperatures met discharge criteria. The impact of hypothermia on SSI rates will continue to be measured over a twelve month period.

## Conclusion

The project achieved its objective by improving awareness and knowledge of hypothermia and SSI’s through bright signage, computer screen savers and increased use of active warming techniques. The new knowledge gained is that hypothermia prevention and management for the surgical patient needs to be ongoing to ensure that the patient’s risk of SSI’s is minimised as much as possible.

## Disclosure of interest

None declared.

